# Immunoglobulin A Vasculitis Associated With COVID-19 Infection Successfully Treated With Corticosteroid Regimen Without Relapse

**DOI:** 10.7759/cureus.28447

**Published:** 2022-08-26

**Authors:** Samantha Davis, Arjun Chandra, Sabeen Sidiki, Aya Abugharbyeh, Nezam Altorok

**Affiliations:** 1 Internal Medicine, The University of Toledo, Toledo, USA; 2 Rheumatology, Internal Medicine, The University of Toledo, Toledo, USA

**Keywords:** coronavirus disease (covid-19), corticosteroid treatment, vasculitis, covid-19, iga vasculitis

## Abstract

Immunoglobulin A (IgA) vasculitis is an autoimmune disease associated with bacterial and viral infections that typically presents with palpable purpura, arthralgia, abdominal pain, and renal involvement. Coronavirus disease 2019 (COVID-19) infection has been found to trigger numerous autoimmune and rheumatologic conditions, including IgA vasculitis. We report a patient who had a COVID-19 infection and then two weeks later developed severe abdominal pain, nausea, emesis, diarrhea, hematochezia, palpable purpura, and arthralgia. Skin biopsy revealed deposition of IgA and C3 complement granular deposition with fibrinogen deposition in superficial dermal vessel walls consistent with IgA vasculitis. The patient was treated with intravenous methylprednisolone followed by oral prednisone with significant improvement and no relapse after tapering and discontinuing steroids in six weeks. This case of biopsy-proven IgA vasculitis precipitated by active COVID-19 infection demonstrates the ability of COVID-19 infection to induce IgA vasculitis and its response to corticosteroid treatment.

## Introduction

Immunoglobulin A (IgA) vasculitis is an autoimmune disease that is characterized clinically by the presence of palpable purpura, arthralgia, abdominal pain, and renal involvement [1]. Since the beginning of the coronavirus disease 2019 (COVID-19) pandemic, numerous autoimmune and rheumatologic diseases have been reported after COVID-19 infection, such as systemic lupus erythematosus, multiple sclerosis, and multisystem inflammatory autoimmune disease [2]. It is known that IgA vasculitis can develop after viral infections, including coxsackievirus, adenovirus, and hepatitis A and B, thus supporting the probable causal relationship between COVID-19 infection and IgA vasculitis [1]. Several mechanisms through which COVID-19 infection can lead to the development of IgA vasculitis have been proposed, as discussed below. Here, we present another case of IgA vasculitis that manifested after COVID-19 infection and was successfully treated with a six-week course of tapered corticosteroids without relapse.

## Case presentation

A 33-year-old male with a history of type 2 diabetes mellitus who tested positive for COVID-19 infection two weeks prior to presentation and had one week of illness presented with diffuse, severe abdominal pain, nausea with non-bilious, non-bloody emesis, diarrhea, and hematochezia. Prior to the development of his gastrointestinal symptoms, the patient had been experiencing worsening erythematous and painful lesions on the lower extremities with extension to the pelvis and buttocks bilaterally over the course of one week. His primary care provider had concerns about vasculitis and placed him on prednisone for two days, which resulted in some improvement. The patient also noted arthralgia in his knees, ankles, and feet. He did not have any urinary symptoms, fever, or chills on presentation. The patient denied sick contacts, recent travel, consumption of undercooked food or food prepared outside the home, and personal or family history of inflammatory bowel disease.

**Figure 1:**
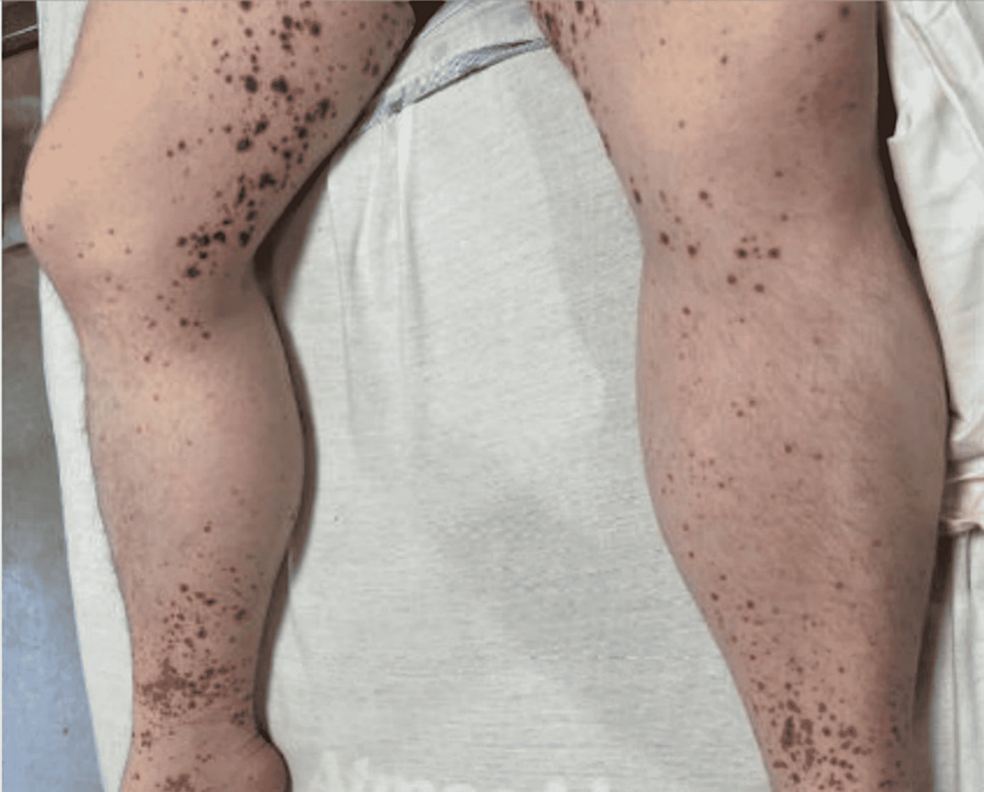
Palpable purpuric rash in patient’s bilateral lower extremities.

Initial physical examination revealed an afebrile and hemodynamically stable patient with diffuse severe abdominal tenderness to palpation, palpable purpura of the bilateral lower extremities with extension to the pelvis and buttocks (Figure 1), and petechiae noted in the bilateral upper extremities.

Outpatient workup before current hospitalization revealed the following: complete blood count with differential was unremarkable. The patient had hyperglycemia and mildly elevated alanine transaminase at 60 U/L (reference range 0-40 U/L) on his complete metabolic panel. Autoimmune workup revealed negative anti-nuclear antibody screening test, antineutrophil cytoplasmic antibodies (ANCA), anti-chromatin immunoglobulin G antibodies, anti-smith antibodies, and antinuclear ribonucleoprotein antibodies. Total complement level, C3 complement, and C4 complement were within normal limits. Chest X-ray was unremarkable.

Inpatient laboratory findings included leukocytosis with neutrophilic predominance likely secondary to recent corticosteroid administration and elevated D-dimer at 808 ng/mL (reference range less than 255 ng/mL). Alanine transaminase normalized during his stay, while aspartate aminotransferase, alkaline phosphatase, blood urea nitrogen, and creatinine were unremarkable. Lipase was within normal limits. Inflammatory markers were elevated with C-reactive protein at 1.6 mg/dL (reference range 0-0.744 mg/dL) and erythrocyte sedimentation rate at 22 mm/hour (reference range 0-15 mm/hour). Ferritin and haptoglobin were within normal limits. A full coagulation profile was not investigated during his stay. Urinalysis revealed glucosuria, proteinuria, and ketonuria; microscopic urine studies were negative for red blood cells and white blood cells. Additional studies such as albumin/creatinine ratio, protein/creatinine ratio, and 24-hour protein collection were not performed during his inpatient stay. To work up the positive screens, antinuclear antibodies via immunofluorescence with reflex and dsDNA by *Crithidia luciliae* were negative. IgA, C3A, and C5A levels were not performed. COVID-19 polymerase chain reaction testing was positive with positive immunoglobulin G antibodies against COVID-19, indicative of recent infection. *Mycoplasma pneumoniae* immunoglobulin M antibodies were negative. The gastrointestinal stool panel was negative for common viral and bacterial etiologies. Stool ova and parasite testing were negative for *Cryptosporidium *and *Giardia*. A punch biopsy of the right lower extremity thigh was performed; direct immunofluorescence revealed granular deposition of IgA and C3 complement with homogeneous deposition of fibrinogen within the walls of many superficial dermal vessels which is a pattern consistent with IgA vasculitis. Computed tomography (CT) angiogram of the chest was unremarkable. CT angiogram of the abdomen and pelvis was negative for acute pathology with a notable patent celiac and superior mesenteric artery without thrombus. The patient underwent esophagogastroduodenoscopy (EGD) and colonoscopy as a workup for his hematochezia. EGD revealed mild patchy gastritis with a single erythematous erosion in the prepyloric stomach; biopsies taken were negative for *Helicobacter pylori*. Colonoscopy revealed erythematous mucosa in the proximal transverse colon; biopsy from this site was unremarkable.

Throughout his hospitalization, the patient was treated with a brief period of oxygen supplementation due to low saturation via nasal cannula at 2 L/minute with later improvement in oxygen parameters to room air, intravenous hydration, and diet advancement as tolerated. Gastroenterology was consulted for hematochezia, ultimately performing EGD and colonoscopy. The patient was started on methylprednisolone sodium succinate 80 mg intravenously daily, receiving a three-day course. The patient improved on corticosteroid treatment. He was transitioned to oral prednisone 60 mg daily at discharge. At the outpatient follow-up a week later, the patient noted his skin lesions were healing. He was then planned for a slow taper of prednisone with 50 mg daily for two weeks, followed by 40 mg daily for two weeks with a follow-up in four weeks to assess response. At the next follow-up in five weeks, the patient had no more skin lesions and his old lesions resolved completely with no scars. Steroid treatment was discontinued with no relapse at three months.

## Discussion

We report the case of a patient with COVID-19 infection who subsequently developed biopsy-proven IgA vasculitis that was successfully treated with a steroid regimen for six weeks with no relapse.

One mechanism through which COVID-19 infection can lead to the development of IgA vasculitis may be due to an inappropriate type 2 T-helper (Th2) response to the virus [3]. Classically, in response to a novel infectious agent, naïve T-helper cells (Th0) cause the immune system to mount either a type 1 T-helper (Th1) response or Th2 response. While a Th1 response in immunocompetent individuals is the default response to intracellular pathogens, such as viruses or bacteria, and is mediated by macrophages and cytotoxic T cells, a Th2 response is directed against extracellular pathogens such as helminths, and is mediated by eosinophils, basophils, mast cells, and B-cells. Roncati et al. have shown that severe acute respiratory syndrome coronavirus 2 (SARS-CoV-2) can cause the immune system to mount a Th2 response instead of a Th1 response, resulting in the activation of B cells and the formation of antibodies [3,4]. Due to a high antigen load, a type 3 hypersensitivity reaction can occur in patients with COVID-19 with the formation of antigen-antibody complexes. Deposition of these immune complexes in blood vessels is accompanied by activation of the complement cascade and release of complement anaphylatoxins C3a and C5a, leading to the development of systemic vasculitis [3].

Another mechanism through which COVID-19 infection can lead to the development of IgA vasculitis is through the induction of endothelial injury either by direct viral infection of endothelial cells via angiotensin-converting enzyme-2 receptors or indirectly through inflammation produced by the host’s immune response [1,5]. Varga et al. have shown the presence of inflammatory cells and SARS-CoV-2 viral components within the vascular endothelium of the heart, lungs, kidneys, and small bowel obtained from post-mortem biopsies of decedents with COVID-19 [6]. Additionally, endothelial cell injury can be caused by neutrophil extracellular traps (NETs), whose primary role is to clear infections by trapping microorganisms [6,7]. The excessive NET release has been implicated in the pathogenesis of COVID-19, thus supporting the role of endothelial cell injury caused by NETs in the development of IgA vasculitis after COVID-19 infection [8].

In addition to our case, a literature review of other cases in 2020-2021 of COVID-19 infection-induced IgA vasculitis was performed (Table 1). We report a 33-year-old male patient who developed COVID-19-induced IgA vasculitis with cutaneous and gastrointestinal involvement successfully treated with corticosteroids. Most of the cases feature a male predominance with a significant portion having renal involvement and successful treatment with corticosteroids [3,9-20]. Our case adds to this growing body of literature. As there are yet to be many female cases reported of COVID-19-induced IgA vasculitis, this may be an area of further investigation.

**Table 1:** Reported cases of COVID-19 infection induced IgA vasculitis during 2020-2021. COVID-19: coronavirus disease 2019; IgA: immunoglobulin A; NSAIDs: non-steroidal anti-inflammatory drugs

Authors	Year	Case Description	Reference
Suso et al.	2020	A 78-year-old male patient with a history of hypertension, dyslipidemia, aortic stenosis, and bladder cancer had COVID-19 pneumonia three weeks prior to developing IgA vasculitis with nephritis. He was successfully treated with corticosteroids and rituximab	[9]
Allez et al.	2020	A 24-year-old male patient with a history of Crohn’s disease was simultaneously diagnosed with COVID-19 infection and IgA vasculitis. He was treated with corticosteroids and enoxaparin	[10]
Sandhu et al.	2020	A 22-year-old male patient with no significant history was diagnosed with COVID-19 infection two days after the development of IgA vasculitis with renal involvement. He was treated with corticosteroids and mycophenolate mofetil	[11]
Mousavi et al.	2020	A six-year-old male patient with no significant history was clinically diagnosed with COVID-19 infection two days after the development of IgA vasculitis with renal involvement. He was treated with corticosteroid, ibuprofen, antibiotics, hydroxychloroquine, and cyclophosphamide; unfortunately, the patient expired due to critical illness	[12]
Hoskins et al.	2020	A two-year-old male patient with no significant history was simultaneously diagnosed with COVID-19 infection and IgA vasculitis. He was treated with corticosteroids and low-molecular-weight heparin	[13]
Nakandakari Gomez et al.	2020	A four-year-old female patient with no significant history had COVID-19 infection one month prior to the development of IgA vasculitis. The patient also had strongyloidiasis. She was treated with corticosteroids, metamizole, piperazine, antibiotics, ivermectin, and omeprazole	[14]
Jedlowski & Jedlowski	2021	A 70-year-old male patient with no significant history was simultaneously diagnosed with COVID-19 infection after the development of IgA vasculitis with renal involvement within one week of exposure. He was treated with corticosteroids initially, lost to follow-up, and re-started on corticosteroids	[3]
AlGhoozi et al.	2021	A four-year-old male patient with no significant history recovered from COVID-19 infection 37 days prior to the development of IgA vasculitis with renal involvement. The patient was treated with as-needed acetaminophen/paracetamol	[15]
Li et al.	2021	A 30-year-old male patient with no prior medical history was simultaneously diagnosed with COVID-19 infection and IgA vasculitis with renal involvement. The patient was successfully treated with corticosteroids	[16]
Jacobi et al.	2021	A three-year-old male patient with a history of surgically corrected Hirschsprung disease was clinically diagnosed with asymptomatic COVID-19 infection, with exposure to family members who tested positive two days prior and developed IgA vasculitis. The patient was treated with corticosteroids	[17]
Barbetta et al.	2021	A 62-year-old male patient with no significant history was diagnosed with COVID-19 pneumonia and then 10 days later developed IgA vasculitis with renal and gastrointestinal involvement. He was treated with hydroxychloroquine, lopinavir/ritonavir, and corticosteroids	[18]
Falou et al.	2021	An eight-year-old male patient with no known history was diagnosed with asymptomatic COVID-19 infection and then three days later developed IgA vasculitis. He was treated with NSAIDs and acetaminophen/paracetamol	[19]
El Hasbani et al.	2021	A 16-year-old male patient with no known history was diagnosed with COVID-19 infection two days prior to the development of IgA vasculitis. He was successfully treated with corticosteroids	[20]

## Conclusions

We present a case of biopsy-proven IgA vasculitis in a patient who was recently diagnosed with COVID-19 infection. The development of IgA vasculitis has been linked to infectious triggers by multiple bacterial and viral organisms. Multiple mechanisms have been proposed to explain the development of COVID-19-induced IgA vasculitis. This case of biopsy-proven IgA vasculitis associated with COVID-19 infection was successfully treated with six weeks of tapered corticosteroids alone and achieved resolution of symptoms and skin lesions without relapse.
